# The Red Cross Red Crescent Health Information System (RCHIS): an electronic medical records and health information management system for the red cross red crescent emergency response units

**DOI:** 10.1186/s13031-024-00585-6

**Published:** 2024-04-08

**Authors:** Felix Holl, Lauren Clarke, Thomas Raffort, Elvire Serres, Laura Archer, Panu Saaristo

**Affiliations:** 1 DigiHealth Institute, Neu-Ulm University of Applied Sciences, Wileystr. 1, 89231 Neu-Ulm, Germany; 2 German Red Cross, Berlin, Germany; 3 International Federation of the Red Cross and Red Crescent Societies, Geneva, Switzerland; 4 World Health Organization, Geneva, Switzerland; 5 International Federation of the Red Cross and Red Crescent Societies, Budapest, Hungary

**Keywords:** Disaster medicine, Emergency care information systems, International federation of red cross and red crescent societies, Electronic medical record, Emergency medical teams, Minimum data set

## Abstract

**Background:**

The Red Cross and Red Crescent Movement (RCRC) utilizes specialized Emergency Response Units (ERUs) for international disaster response. However, data collection and reporting within ERUs have been time-consuming and paper-based. The Red Cross Red Crescent Health Information System (RCHIS) was developed to improve clinical documentation and reporting, ensuring accuracy and ease of use while increasing compliance with reporting standards.

**Case presentation:**

RCHIS is an Electronic Medical Record (EMR) and Health Information System (HIS) designed for RCRC ERUs. It can be accessed on Android tablets or Windows laptops, both online and offline. The system securely stores data on Microsoft Azure cloud, with synchronization facilitated through a local ERU server. The functional architecture covers all clinical functions of ERU clinics and hospitals, incorporating user-friendly features. A pilot study was conducted with the Portuguese Red Cross (PRC) during a large-scale event. Thirteen super users were trained and subsequently trained the staff. During the four-day pilot, 77 user accounts were created, and 243 patient files were documented. Feedback indicated that RCHIS was easy to use, requiring minimal training time, and had sufficient training for full utilization. Real-time reporting facilitated coordination with the civil defense authority.

**Conclusions:**

The development and pilot use of RCHIS demonstrated its feasibility and efficacy within RCRC ERUs. The system addressed the need for an EMR and HIS solution, enabling comprehensive clinical documentation and supporting administrative reporting functions. The pilot study validated the training of trainers’ approach and paved the way for further domestic use of RCHIS. RCHIS has the potential to improve patient safety, quality of care, and reporting efficiency within ERUs. Automated reporting reduces the burden on ERU leadership, while electronic compilation enhances record completeness and correctness. Ongoing feedback collection and feature development continue to enhance RCHIS’s functionality. Further trainings took place in 2023 and preparations for international deployments are under way. RCHIS represents a significant step toward improved emergency medical care and coordination within the RCRC and has implications for similar systems in other Emergency Medical Teams.

## Background

The Red Cross and Red Crescent Movement (RCRC) has developed specialized Emergency Response Units (ERUs) to support its humanitarian activities in disaster response. ERUs are pre-packaged and self-sufficient humanitarian aid modules that can be rapidly deployed to disaster-affected areas to provide essential services such as water supply, sanitation, medical care, and shelter. They are designed to be flexible and adaptable to different types of emergencies, and can be deployed as a standalone unit or integrated into a larger response effort. The ERUs are operated by National Red Cross and Red Crescent Societies coordinated by the International Federation of Red Cross and Red Crescent Societies (IFRC). ERUs are equipped with trained personnel, specialized equipment and materials, and are able to provide support to affected communities within 72 h of deployment.

ERU Health modules are an important component of the RCRC disaster response. Health modules with different configurations and capabilities are available. The emergency clinic module (fixed or mobile) can provide outpatient services. The fixed clinic can provide initial emergency care of injuries and other significant health care needs for adults and children. Mobile emergency clinic can provide primary health care and urgent services to adults and children within hard-to-reach communities [1]. The emergency hospital module can provide acute medical care, general and obstetric surgery for trauma and other major medical conditions [2]. The RCRC ERUs are the equivalent of Type 1 (fixed and mobile) and Type 2 facilities in the Emergency Medical Teams (EMT) classification from the World Health Organization (WHO), which is regulated through the Red Channel Agreement [3].

The key feature of EMTs is their classification by the World Health Organization (WHO) to ensure treatment quality and logistical operational capability. In the event of an incident, EMTs are requested and coordinated by the respective government of the affected country. The global ‘EMT Initiative’ led by the WHO is a consequence of the earthquake disaster in Haiti in 2010, where many emergency teams were deployed in an uncoordinated manner without quality control. The goal of the initiative is to improve the quality and availability of emergency medical care by increasing the number of well-trained and well-equipped emergency medical teams [4]. The EMT initiative also aims to promote the integration of emergency medical services into national health systems and to improve the coordination of emergency medical care at the international level.

As part of the EMT initiative, WHO has established a Minimum Data Set (MDS) for Emergency Medical Teams (EMT) to facilitate the reporting and monitoring of EMTs [5]. The items of the MDS were defined in a working group [6]. The MDS is a set of standardized data elements that EMTs are required to report to the Emergency Medical Team Coordination Cell (EMTCC) on a daily basis. The EMTCC shares the MDS reports in aggregated form with the ministry of health of the affected country and with other relevant stakeholders. This data is used to track the deployment and performance of EMTs and to identify areas for improvement.

The MDS for EMTs is intended to be used by EMTs, national health authorities, and international organizations to improve the quality and availability of emergency medical care. By providing a standardized set of data elements, the MDS allows for the comparison of different EMTs and the identification of best practices.

Standardization of data collection and reporting within EMTs is challenging [7]. In past deployments, the Red Cross Red Crescent Type 1 and Type 2 facilities have collected data by hand using paper-based form and Excel spreadsheets. This process can be laborious, time-consuming, and often inaccurate.

## Case presentation

The Red Cross Red Crescent Health Information System (RCHIS) has been designed to improve clinical document and reporting within the RCRC Health ERUs, to ensure that reporting is accurate and easy to complete, increasing compliance with the EMT-MDS reporting.

**Fig. 1:**
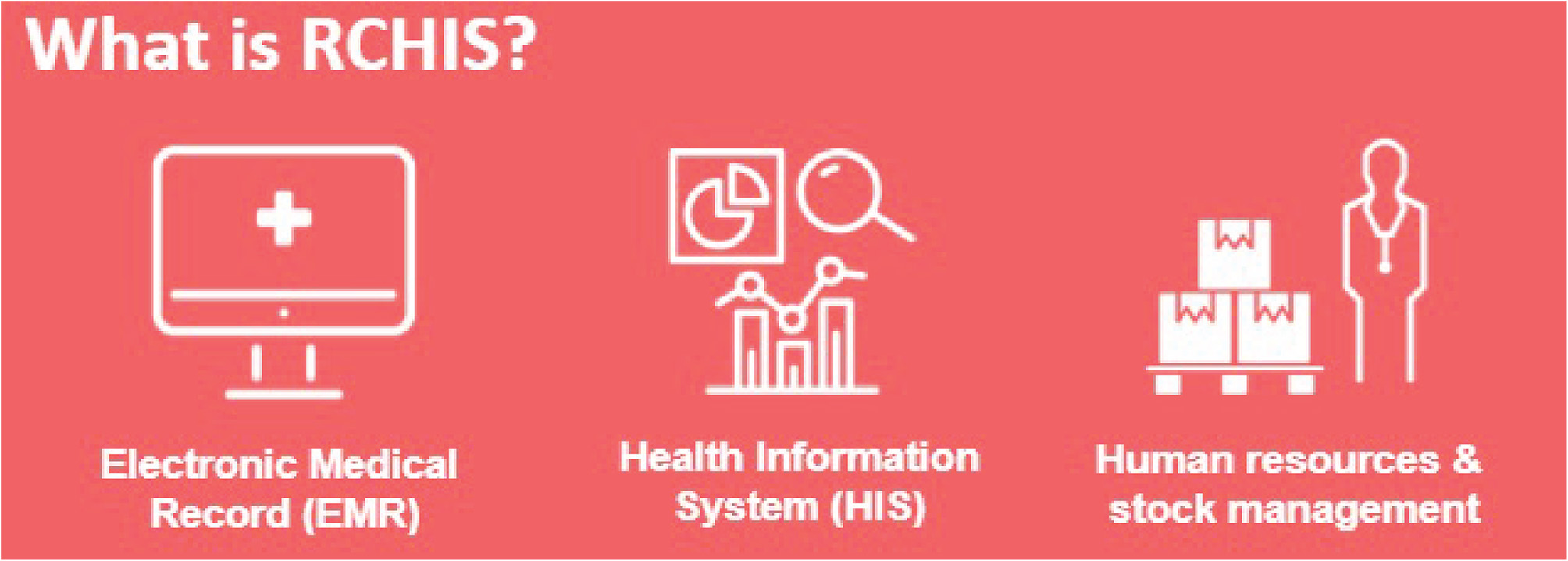
Components of RCHIS

RCHIS is an Electronic Medical Record (EMR) and Health Information System (HIS) that has been purpose built for use by RCRC ERUs and enables live operational updates (see Fig. 1). The aim of this case presentation is to share the main lessons learned from the development and pilot use of RCHIS to inform development and implementation of similar systems in other EMTs.

### Concept

The need for an EMR and HIS for the ERUs was identified in 2015 and a landscape analysis was conducted to examine existing solutions and assess their fit for the ERU context. The analysis yielded that no existing tool are fit for the purpose and the need to develop a purpose-build tool.

The development was done in an iterative process as a collaboration between IFRC Emergency Health team, IFRC IT department, RCRC national societies and an external software development company. A series of workshops were conducted to design the different modules of RCHIS, including bringing in expert advice from National Societies.

**Fig. 2:**
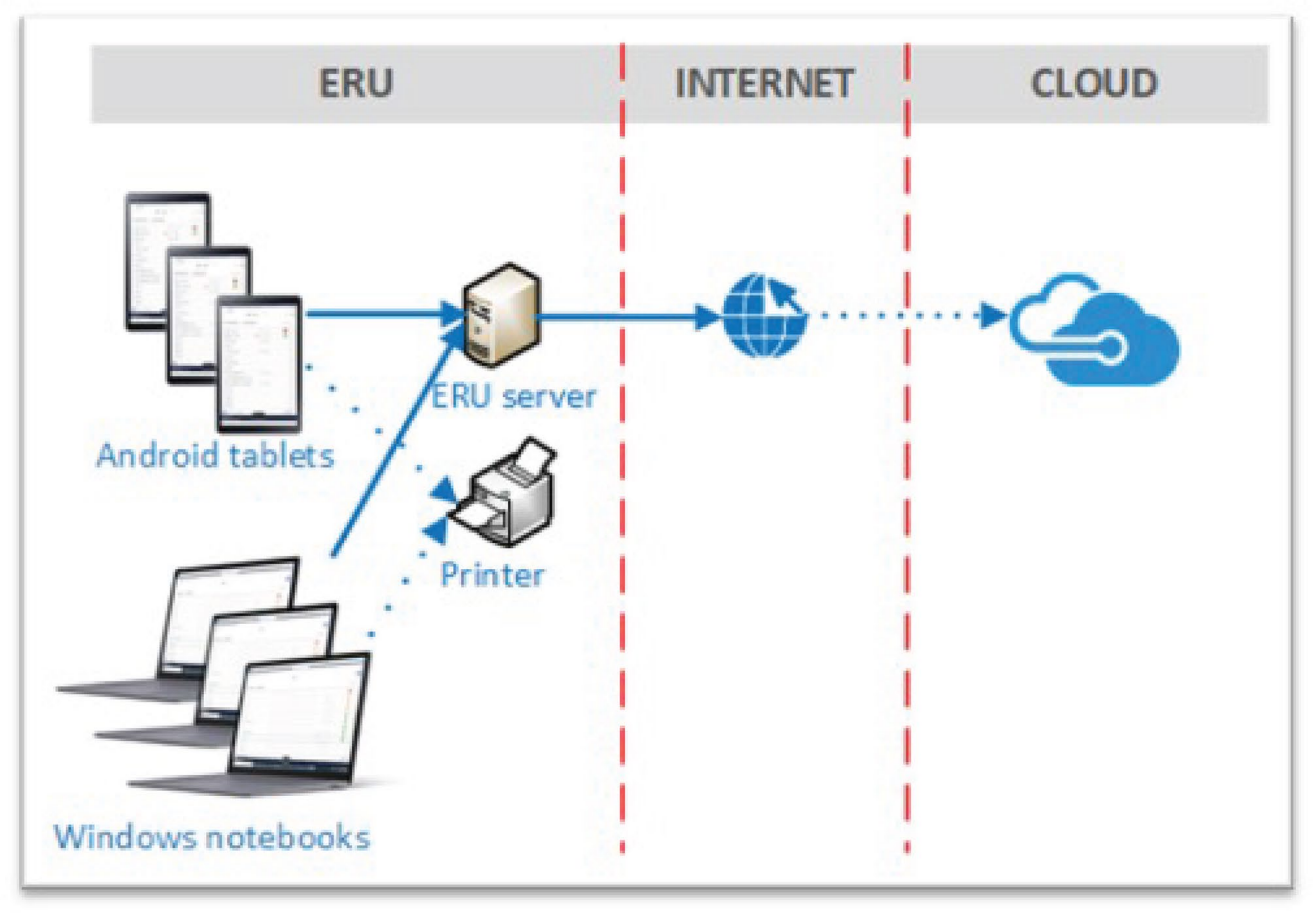
Architecture of RCHIS

RCHIS can be run on Android tablet or Windows Laptop and works on- and offline. The data is synced and stored on a secure Microsoft Azure cloud. In addition, a local ERU server is deployed to enable synchronization between the end-user devices in case of interruptions to the internet connection. Figure 2 gives an overview of the technical architecture of RCHIS. The components within an ERU are Android tablets and Windows laptops, on which the RCHIS application is running and are used by the end users and a local network, wired and wireless. Additional optional components are printers with a network connection and a ERU server, that serves as a local synchronization. The ERU network is connected to the internet. Data from the end user devices and the local ERU server are send to the cloud-based Microsoft Azure servers.

**Fig. 3:**
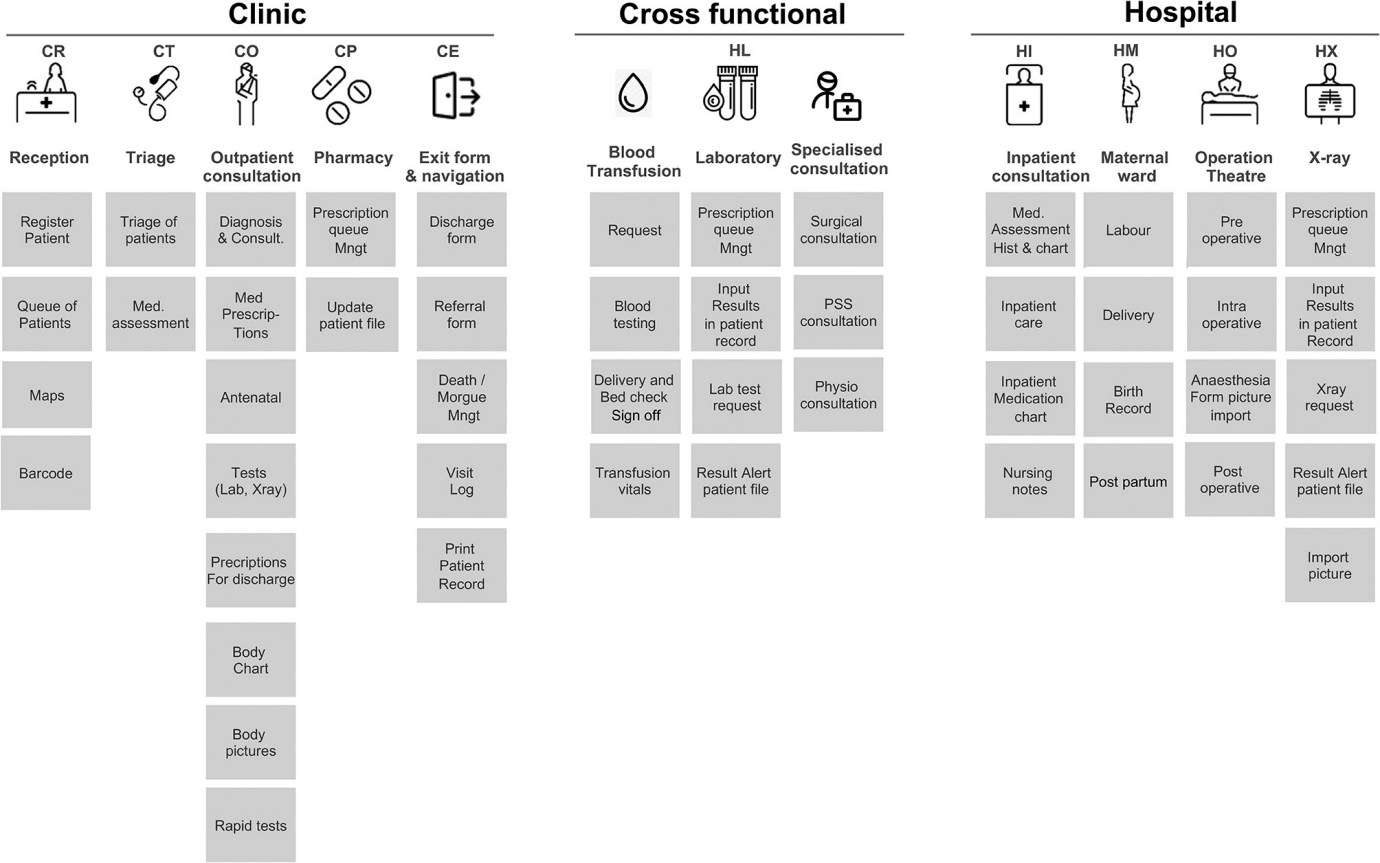
Functional map of RCHIS

The functional architecture is designed to cover all clinical functions of both ERU clinics and hospitals (see Fig. 3). Elements required for mandatory reporting were added into the application as dropdown and selection elements, while many other elements consist of free text fields. The reasoning behind using free text fields was to streamline the user experience and ensure that the application is not preventing clinicians from doing their work when using the application.

### Pilot

The first pilot of both the end user-training and use of RCHIS was done May 2022 with the Portuguese Red Cross (PRC) [8]. First, a three-day, in-person super user training was held with 13 participants: nine first aid volunteers, two nurses, and two medical doctors; seven delegates had experience using an EMR. These super users served as trainers for staff at the pilot.

The pilot occurred with the PRC at the Peregrinação de Fátima. The Peregrinação de Fátima is the annual to the pilgrimage to the Sanctuary of Fátima in Central Portugal, which attracts large groups from all over Portugal and beyond. In 2022, there where 200,000 people were in attendance. The PRC was part of a wider coordination cell with the civil defense authority, who required live reporting from the three clinics PRC had set up. During the four-day pilot, 77 user accounts and 243 patient files were created. The delegates shared feedback directly and through a survey. 88% stated that RCHIS was very easy to use with the majority of delegates requiring less than 30 min of training. 95% of delegates stated that they had sufficient training to use RCHIS to its full extent. The civil defense authority was able to utilize the real-time reporting to assist in their operational response.

### Training

The training of both clinical and managerial end-users of RCHIS has been designed as a training of trainers’ approach. Super users and expert users, which will train end-user prior to and during deployment as well as will serve as first level support during a deployment, are trained in a 4 day in-person training. The training consists of mixture of instructive lectures and simulations exercises. After a first pilot of the training during the RCHIS pilot in Portugal, a first dedicated training was held in September 2022.

### Reporting

In addition to clinical documentation, reporting is another main function of RCHIS. This includes both the automated generation of the WHO MDS daily reporting form and additional reporting for operational and donor reporting.

Especially the daily MDS reporting of all cases, which is required by an WHO mandated EMT mission, to the EMTCC is very labor intensive and error-prone if done based off paper-based documentation. RCHIS can help to reduce the time necessary to generate and share the daily MDS report significantly [9].

## Discussion and conclusions

After the need for an EMR and HIS system for the RCRC ERUs has been identified, the initial concept and design phase of RCHIS required significant effort by key stakeholders within the RCRC movement to ensure best fit of this purpose-built solution. All clinical functions of the clinical ERUs can be documented with RCHIS and administrative functions can be supported with automated reporting functions such as the MDS generation.

The first pilot of RCHIS in a domestic setting with a type 1 fixed clinical equivalent was successful and served as the first test of the feasibility of RCHIS. The pilot was successful from both a technical and organizational perspective and included a first pilot of the training concept. The training of trainer concept for RCHIS worked and delegates trained by those training during the first training were able and comfortable in using RCHIs. Following the pilot, RCHIS is continuously being used in a domestic setting in Portugal. Additional end-user feedback on the tool and newly developed features is being collected during those uses. In addition, the domestic use of RCHIS by RCRC societies in other countries is in preparation. The domestic pilot is an important step towards the use of RCHIS in international disaster responses.

Improved clinical documentation is beneficial for patient safety and quality of care within the ERUs. Automated reporting, such as the automatic generation of the daily MDS reporting form can considerably lessens the burden of the ERU leadership. In addition, compared to the manual compilation of the MDS based off paper-based records utilizing tally sheets, the automated development of the daily MDS report based off the EMR data improves record completeness and correctness.

Detailed preparations and SOPs will be needed to meet the challenges of using an electronic documentation system like RCHIS in the context of EMTs, especially in view of the short preparation times for international operations. It is essential to ensure interoperability with digital coordination tools for the EMTCC that are currently being designed and developed. Having procedures in place to ensure appropriate processes for obtaining patient consent is key. Long-term, the ability to share data with other EMTs will be beneficial to ensure continuity of care during major emergencies.

Additional super- and expert-user trainings were conducted in 2023 and preparations for first international deployments with clinical ERUs are underway.

## Data Availability

Data sharing is not applicable to this article as no datasets were generated or analysed during the current study.

## Declarations

### Authors’ information

Felix Holl has training in medical informatics and global health. He is a postdoctoral scholar at the DigiHealth Institute, Neu-Ulm, and a health informatics consultant for German Red Cross. He has served as the coordinator of the Emergency Medical Teams Coordination Cell in Moldova.

Lauren Clarke is a medical doctor by training and has a background in disaster response. She is the senior officer for medical services in emergencies at the International Federation of Red Cross and Red Crescent Societies.

## Abbreviations

ERU: Emergency Response Unit
EMR: Electronic Medical Record
EMT: Emergency Medical Team
EMTCC: Emergency Medical Teams Coordination Cell
HIS: Health Information System
MDS: Minimum Data Set
RCHIS: Red Cross and Rec Crescent Health Information System
RCRC: Red Cross Red Crescent
WHO: World Health Organization

## References

[1] IFRC, ERU RED CROSS RED, CRESCENT EMERGENCY CLINIC [Internet]. [cited 2023 Feb 21]. Available from: https://go.ifrc.org/deployments/catalogue/health/eru-red-cross-red-crescent-emergency-clinic.

[2] IFRC, ERU RED CROSS RED, CRESCENT EMERGENCY HOSPITAL [Internet]. [cited 2023 Feb 21]. Available from: https://go.ifrc.org/deployments/catalogue/health/eru-red-cross-red-crescent-emergency-hospital.

[3] IFRC & WHO. The Red Channel Agreement - A guidance document regarding the collaboration between IFRC Medical Response Units and the WHO EMT Initiative [Internet]. 2020. Available from: https://www.jrc.or.jp/international-delegates/information/pdf/The%20Red%20Channel%20Agreement%20-%20A%20guidance%20document%20regarding%20the%20collaboration%20between%20IFRC%20Medical%20Response%20Units%20and%20the%20WHO%20EMT%20Initiative%20May%2018%202021%20FINAL.pdf.

[4] Hamilton ARL, Södergård B, Liverani M. The role of emergency medical teams in disaster response: a summary of the literature. Nat Hazards. 2022;110:1417–1426. 10.1007/s11069-021-05031-x.

[5] Kubo T, Yanasan A, Herbosa T, Buddh N, Fernando F, Kayano R. Health Data Collection Before, During and After Emergencies and Disasters—The Result of the Kobe Expert Meeting. Int J Environ Res Public Health. 2019;16(5):893. 10.3390/ijerph16050893. 10.3390/ijerph16050893 PMC6427760 30871037

[6] Benin-Goren O, Kubo T, Norton I. Emergency Medical Team Working Group for Minimum Data Set. Prehospital Disaster Med. 2017;32(S1):S96–S96. 10.1017/S1049023X17002473.

[7] Jafar AJN (2020). Disaster documentation: improving medical information-sharing in sudden-onset disaster scenarios. Third World Q.

[8] Clarke L, Holl F, Raffort T, Serres E, Órfão G, Simões J (2022). Deploying the Red Cross Red Crescent Health Information System (RCHIS): a pilot with the Portuguese Red Cross in a Red Cross Emergency Clinic (RCEC) Equivalent to a type 1 fixed clinic. Prehosp Disaster Med.

[9] Clarke L, Holl F, Raffort T, Serres E, Archer L, Saaristo P (2022). From 2 hours to 2 seconds: using the Red Cross Red Crescent Health Information System (RCHIS) to complete the Emergency Medical teams - Minimum Data Set Reporting. Prehosp Disaster Med.

